# Modulating cell adhesion and infiltration in advanced scaffold designs based on PLLA fibers with rGO and MXene (Ti_3_C_2_T_*x*_)

**DOI:** 10.1016/j.mtbio.2025.101785

**Published:** 2025-04-21

**Authors:** Martyna Polak, Krzysztof Berniak, Piotr K. Szewczyk, Joanna Knapczyk-Korczak, Mateusz M. Marzec, Muhammad Abiyyu Kenichi Purbayanto, Agnieszka M. Jastrzębska, Urszula Stachewicz

**Affiliations:** a Faculty of Metals Engineering and Industrial Computer Science, AGH University of Krakow, Al. A. Mickiewicza 30, Krakow, 30-059, Poland; b Academic Centre for Materials and Nanotechnology, AGH University of Krakow, Al. A. Mickiewicza 30, Krakow, 30-059, Poland; c Warsaw University of Technology, Faculty of Mechatronics, św. A. Boboli 8, Warsaw, 02-525, Poland

**Keywords:** PLLA, MXenes, rGO, Osteoblast infiltration, Surface charge, Focal adhesions, Cluster analysis

## Abstract

The development of electrospun scaffolds that support cell adhesion and infiltration remains a critical challenge in tissue engineering. In this study, we investigate the influence of two-dimensional (2D) fillers—reduced graphene oxide (rGO) and MXene (Ti_3_C_2_T_*x*_)—incorporated into poly(L-lactic acid) (PLLA) electrospun fibers on their properties and osteoblast responses. The presence of fillers modified fiber arrangement and created varying inter-fiber spacing due to surface charge repulsion and agglomeration. Importantly, surface potential measurements via Kelvin probe force microscopy (KPFM) of PLLA fibers show a significant shift caused by the incorporation of Ti_3_C_2_T_*x*_ to ∼400 mV compared to ∼50 mV for rGO. *In vitro* tests indicate that rGO-modified scaffolds support osteoblast infiltration up to ∼100 μm, unlike PLLA fibers, which limit cell infiltration to a maximum of ∼70 μm. However, Ti_3_C_2_T_*x*_ promotes even deeper (∼120 μm) and more uniform cell's infiltration due to changes in scaffold architecture. High-resolution confocal imaging confirmed that PLLA-Ti_3_C_2_T_*x*_ fosters larger, elongated adhesion site clusters of cells, whereas rGO increases cell's adhesion site density in relation to PLLA scaffolds without any filler. Our findings highlight the distinct roles of rGO and Ti_3_C_2_T_*x*_ in modulating scaffold geometry, mechanical behavior, and cellular interactions. Tailoring the composition and distribution of conductive fillers in fibers offers a promising strategy for optimizing scaffold performance in tissue engineering applications.

## Highlights

• MXenes promote larger, elongated adhesion sites, affecting cell adhesion.
• MXenes enhance cell infiltration, improving scaffold performance over rGO.
• Surface potential of fibers shifts with the addition of conductive fillers.
• MXenes and rGO alter fiber structure, with MXenes forming smaller clusters.

## Graphical abstract

**Figure:**
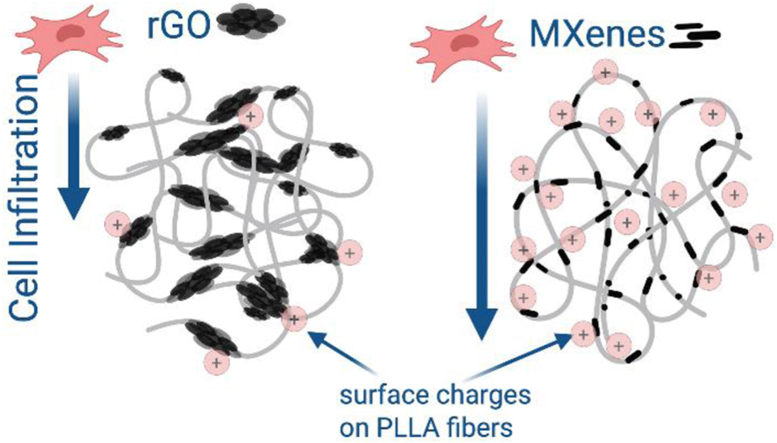

## 1 Introduction

The design of advanced tissue engineering scaffolds focuses on developing materials mimicking the extracellular matrix (ECM) to create a supportive environment for tissue regeneration and expose cells to stimulating conditions. These highly complex materials, with properties defined during their fabrication, are not only intended to regenerate tissue by stimulating cells but are also utilized in other applications [1]. These include drug delivery, cancer treatment, the creation of organoids used as platforms to simulate specific organs, and studying and modeling diseases [[2], [3], [4]]. However, it is worth noting that fundamental studies on novel scaffolds often miss the direct distinction between the material's single property and the triggered biological response. For instance, research on electrospun fibers often emphasizes their structural and functional design for specific applications; while these studies highlight the benefits of fibers, they frequently do not discuss challenges like cell migration throughout the scaffolds [5,6]. Limited pore size, dense fiber packing, and insufficient spacing between fibers are common in electrospun scaffolds and significantly hinder cell migration, which is essential for tissue ingrowth, nutrient transport, and long-term scaffold integration [5]. Moreover, cellular adhesion remains the initial cell response to the material, which affects on later tissue development, especially considering signaling pathways essential for early osseointegration and long-term bone healing [[7], [8], [9]]. Focal adhesions, as protein complexes, answer how cells interact with biomaterials [10]. Their size, distribution, and dynamics influence adhesion strength, migration efficiency, and cascade of cellular responses critical for scaffold integration [11]. Therefore, cell adhesion and migration are closely connected. It was reported that cells on nanofiber scaffolds formed larger focal adhesion clusters, enhancing migration speed compared to cells on flat surfaces [12].

Despite numerous advances in scaffold fabrication, many current investigated scaffolds struggle to mimic the electroactive environment of ECM to support sufficient cell infiltration. These limitations hinder scaffold integration and functional tissue regeneration. Our study addresses this gap by exploring how conductive fillers can be used not only to adjust surface charge but also to influence fiber arrangement and inter-fiber spacing, ultimately improving cell-fiber interactions and cells infiltration deep into scaffolds. The key strategy to improve cell infiltration in the electrospun scaffolds is control over the scaffold porosity [5,[13], [14], [15]]. Among the various modifications in the electrospinning process to control scaffold geometry, we recognize the lack of research on composite fiber architecture and the fiber-fiber repellency effect caused by their charged surfaces [[16], [17], [18]]. Recently, we have observed increased interest in developing biomaterials biomimicking electrically active cell environments by incorporating conductive fillers [19,20]. Various cell behavior is influenced by local electric fields due to the usually negatively charged cell membrane [21]. Conductive fillers added to the scaffolds can create the favorable surface charges enhancing the electrostatic responses of cells to materials [22]. However, the effect of their incorporation in electrospun fibers on their architecture and later cell infiltration and adhesion was never addressed. Among the others, two-dimensional (2D) inorganic materials like graphene oxide (GO), reduced graphene oxide (rGO), and transition metal carbides, nitrides, or carbonitrides (MXenes) have lately been extensively studied [[23], [24], [25]]. GO and rGO are investigated graphene derivatives in biomedical applications, attributed to their superior mechanical strength, thermal stability, and flexibility [26]. GO is distinguished by abundant hydrophilic functional groups—hydroxyl, carboxyl, and epoxy—on its surface, whereas rGO exhibits a reduced presence of these groups, resulting in decreased hydrophilicity. The integration of graphene-based nanoparticles into soft biomedical polymers has emerged as a promising strategy in tissue engineering [27,28]. Recent studies have explored the incorporation of rGO into electrospun polymer fibers to develop scaffolds with improved properties for tissue engineering applications [26,29]. For instance, electrospun polyetherimide nanofibers integrated with rGO have shown enhanced conductivity and porosity, which are beneficial for electrochemical sensing electrodes [30]. Moreover, it was reported that rGO in polymer composite supported human mesenchymal stem cell (hMSC) proliferation and osteogenic differentiation. Additionally, these scaffolds showed antimicrobial properties [31,32]. On the other hand, MXenes, derived from MAX phases, invented by Naguib, Gogotsi, and Barsoum, are 2D materials possessing features like high electrical conductivity, large surface area, rich surface chemistry, hydrophilicity, and biocompatibility [33,34]. MXenes, due to their unique properties, have garnered significant attention and show great promise as functionalizing materials for electrospun fibers in various applications like energy storage and conversion, as well as biomedical [23,[35], [36], [37], [38]]. Awasthi et al. showed that polycaprolactone-MXene composite electrospun fibers promoted protein adsorption and biomineralization for pre-osteoblast cells [39].

**Fig. 1:**
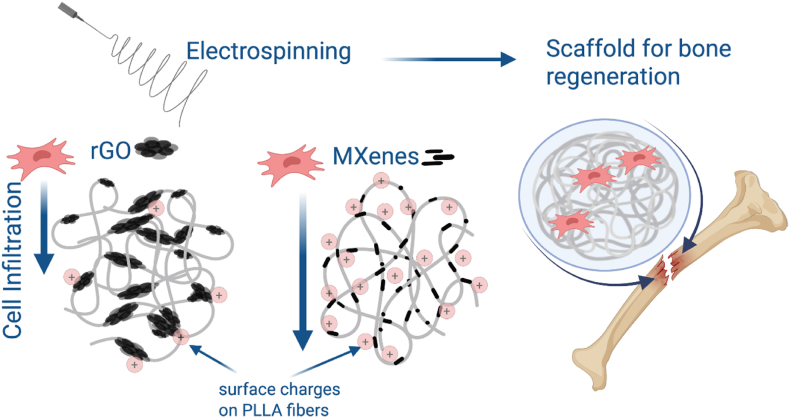
Conceptual scheme of the study on conductive filler effect on scaffold performance and cell responses.

In response to the growing interest in rGO and MXenes due to their unique properties, we address the question of how 2D fillers, rGO, and MXenes – namely, Ti_3_C_2_T_*x*_, influence the surface and mechanical properties of fibers and if it is possible to use the fillers to induce a repelling effect in fibers. Using Kelvin probe force microscopy (KPFM) to evaluate the surface potential, we verify the surface charges with nanoscale precision. Poly(L-lactic acid) (PLLA) was selected as the polymer matrix due to its established biocompatibility, biodegradability, and mechanical properties suitable for bone tissue engineering applications [[40], [41], [42]]. Compared to other biopolymers such as PCL (polycaprolactone) and PLGA (poly(lactic-co-glycolic) acid), PLLA offers higher mechanical strength and a slower degradation rate, making it particularly advantageous for applications requiring long-term structural support, such as bone regeneration [43]. For the first time, we correlate the incorporation of fillers with cell adhesion and infiltration. Our analysis uniquely examines focal adhesion points on fiber regions with 2D materials, revealing clear differences in adhesion patterns and density. We show that the problem of limited cell migration can be solved by incorporating fillers that can, in a controlled manner, change the surface potential of fibers, providing novel insights into scaffold design for biomedical applications. The concept of the study is presented in Fig. 1.

## 2 Materials and methods

### 2.1 Materials

Poly(L-lactide) (PLLA, PURASORB PL18, M_w_ = 221, 000 g mol^−1^, Corbion, The Netherlands) was dried before solution preparation for 2h at 30 °C in a drying oven (POL-EKO-APARATURA, Poland). A polymer solution of 9 wt% concentration was prepared by dissolving PLLA in a mixture of dichloromethane (DCM, analytical standard, Avantor, Poland) and dimethylformamide (DMF, analytical standard, Avantor, Poland) in the ratio of 7:3 w/w. The polymer was dissolved entirely after 3h of stirring at 25 °C, at a constant speed of 400 rpm, using the magnetic stirrer (IKA, Germany) in DCM. We prepared 3 solutions: PLLA, PLLA with reduced graphene oxide (PLLA-rGO), and PLLA with Ti_3_C_2_T*_x_* (PLLA- Ti_3_C_2_T*_x_*). The synthesis of Ti_3_C_2_T_*x*_ was performed from the Ti_3_AlC_2_ MAX phase (Carbon, Ukraine) by the classical acidic aluminum extraction method, as already reported [44]. The standard procedure of PLLA solution preparation requires first dissolving PLLA pellets in DCM, and when PLLA is dissolved, DMF is added. In the case of solutions with fillers, before adding DMF to the PLLA solution, it was used to prepare a homogenized suspension for two samples containing rGO and Ti_3_C_2_T_*x*_. The fillers in the concentration of 16.7 wt% to polymer mass were ultrasonicated in DMF for 2 h using an ultrasonic bath (Sonorex Bandelin, Germany). Next, DMF suspensions were added to PLLA solutions and stirred for 1 h and later ultrasonicated for 1 h. Finally, all solutions were mixed using a vortex with the highest possible vibration frequency (Labnet VX-200 Vortex Mixer, Labnet International, USA) for 2 min before electrospinning. As mentioned, the filler concentration reached 16.7 wt% to ensure equal loading of rGO and Ti_3_C_2_T_*x*_ while maintaining a stable and repeatable fiber formation during electrospinning. It was the highest achievable concentration without causing nozzle clogging or electrospinning discontinuity, ensuring reproducibility of produced scaffolds.

### 2.2 Electrospinning

Electrospinning of all samples was conducted using equipment with a climate control chamber (EC-DIG apparatus, IME Technologies, the Netherlands) at a constant temperature T = 25 °C and relative humidity RH = 40 %. The process required using a stainless-steel nozzle (0.51 mm inner (ID) and 0.82 mm outer diameter (OD), 21G × 1 1/2). The voltage, nozzle-collector distance, and flow rate values are presented in Table 1. The thickness of the mats was controlled by the time of deposition and flow rate to collect the same amount of material for each sample for all the experiments. The conductivity of polymer solutions was measured with a Mettler Toledo Conductometer (Seven Compact S210, Zurich, Switzerland). The conductivity value is an average value from 10 measurements; the error represents the standard deviation.

**Table 1:** Electrospinning parameters and polymer solution conductivity.

Sample	Voltage [kV]	Nozzle-collector distance [cm]	Flow rate [mL/h]	Conductivity [μS/cm]
PLLA	13.5	16	6	1.83 ± 0.1
PLLA-rGO	16.5	16	3.5	11.78 ± 0.22
PLLA-Ti_3_C_2_T_*x*_	19	16	1.2	10.53 ± 0.12

### 2.3 Scanning electron microscopy, energy dispersive spectroscopy (SEM, EDS-SEM)

The morphology of the produced samples was analyzed using scanning electron microscopy (SEM, Merlin Gemini II, ZEISS, Germany). Before imaging, samples were coated with an 8 nm Au layer using a sputter coater (Q150RS, Quorum Technologies, UK). Imaging was conducted at an accelerating voltage of 2–3 kV and a working distance ranging from 3 to 7 mm, using an SE detector. The average diameters (D_f_) were determined by analyzing 100 randomly selected fibers from SEM micrographs using ImageJ software (version 1.53d, USA). To prepare a graph, the kernel smooth distribution in Origin software was used (ver. 2022 9.9 USA). To investigate the distribution of Ti_3_C_2_T*_x_* in PLLA fibers, elemental mapping was performed using energy-dispersive X-ray spectroscopy (EDS, Brucker, Germany). Before EDS analysis, samples were coated with a thin layer of C (approximately 15 nm) using a carbon evaporator (K950 Emitech, Quorum Technologies, UK). Mapping was conducted utilizing a backscattered electron detector for 500 s at an accelerating voltage of 15 kV, a current of 550 pA, and a working distance ranging from 6 to 7 mm.

### 2.4 Contact angle measurement

Contact angle measurement was carried out by depositing 3 μL deionized water droplets (Spring 5UV purification system, Hydrolab, Poland) onto the surface of scaffolds. Images of droplets were captured after 3 s from the deposition, using a Canon camera (EOS 700D, EF-S 60 mm f/2.8 Macro USM zoom lens, Canon, Japan). ImageJ software (version 1.53d, USA) was used to measure contact angles from the images of ten droplets per sample.

### 2.5 Tensile test

Electrospun mats were cut into samples measuring 130 × 70 mm, which were subsequently tested using a tensile machine (20 N cell, Kammrath & Weiss, Germany) at T = 24 °C and RH = 40 %, with an extension rate of 50 μms^−1^. The stress was calculated by dividing the force measured by the machine by the initial cross-sectional area of the electrospun mats. The thickness of the samples was measured in the z-direction at ten different points using a SEM (Phenom ProX Desktop SEM, Thermo Fisher Scientific, USA). To determine the average values for toughness (W), maximum stress (Rm), elongation at maximum stress (Ɛmax), and elongation at failure (Ɛ_f_), five separate measurements were taken for each scaffold sample, and the data were processed using the Integrate function in OriginPro software (ver. 2022 9.9, USA). The curves presented on the graph are the averages of the five tensile tests of each type of sample. Errors are based on the standard deviation.

### 2.6 Filler agglomeration distribution analysis

For each sample, 20 images were captured in transmitted light across different fields of view (confocal laser scanning microscope, Zeiss LSM 900, Germany). The images for all samples were recorded using the same imaging parameters. Agglomeration size detection for Ti_3_C_2_T_X_ and rGO was performed using macros written in ImageJ software (version 1.51, Fiji, USA). The first step of the process involved the detection of fibers. Ti_3_C_2_T_*x*_ or rGO agglomerations were identified exclusively within the areas defined by the previously established fiber mask in the images. Morphological analysis of the identified agglomerates was conducted using ImageJ (version 1.53d, USA). Errors on the graph represent the standard deviation.

### 2.7 Thermal properties (DSC), surface and bulk chemistry analysis (XPS, FTIR)

Thermal properties were analyzed using differential scanning calorimetry (DSC) (DSC 3, Mettler Toledo, Switzerland). The DSC curves present the average values of three independent measurements per sample heated at a rate of 10 Kmin^-1^ from 0 to 230 °C. To identify the functional groups in samples, Fourier transform infrared spectroscopy (FTIR) was used (FTIR, Nicolet iS5, Thermo Fisher, USA) in a range of 400–4000 cm^−1^ with a total number of 64 scans using Ge crystal plate.

Analysis of the surface chemistry of fibers deposited on silicon wafers was performed with X-ray photoelectron spectroscopy (XPS). The XPS analyses were carried out in a PHI VersaProbeII Scanning XPS system using monochromatic Al Kα (1486.6 eV) X-rays focused to a 100 μm spot and scanned over the area of 400 μm × 400 μm. The photoelectron take-off angle was 45° and the pass energy in the analyzer was set to 117.50 eV (0.5 eV step) for survey scans and 46.95 eV (0.1 eV step) to obtain high energy resolution spectra for the C 1s, and O 1s regions. A dual beam charge compensation with 7 eV Ar^+^ ions and 1 eV electrons were used to maintain a constant sample surface potential regardless of the sample conductivity. All XPS spectra were charge referenced to the unfunctionalized, saturated carbon (C-C) C 1s peak at 285.0 eV. The analytical chamber operating pressure was less than 3 × 10^−9^ mbar. Deconvolution of spectra was done using PHI MultiPak software (v.9.9.3). The Shirley method was used to correct the spectrum background.

### 2.8 Kelvin probe force microscopy (KPFM)

Atomic force microscopy (AFM) and KPFM were performed using a CoreAFM system (Nanosurf, Switzerland). For KPFM, conductive HQ:NSC18/Pt tips (MikroMasch, Bulgaria) with a force constant of 2.8 Nm^−1^ and a resonance frequency of 75 kHz were utilized. During KPFM measurements, topographical data were captured simultaneously. Scans covered areas of 100 μm^2^. KPFM data represent the average of 6 separate scan lines taken on top of the fibers. To provide a control for the KPFM signal, measurements from the ITO glass were taken alongside fiber measurements. KPFM measurements were performed in tapping/lift mode with simultaneous topography acquisition, and all scans were conducted in a controlled environment during a single session (RH = 50–65 %, T = 23 °C) to minimize electrostatic artifacts and ensure reproducible surface potential mapping, following similar protocols reported in the literature [37]. Data was analyzed using Gwyddion (v2.56, gwyddion.net) and OriginPro (version 2022 9.9, USA) software.

### 2.9 Zeta potential measurement

Streaming zeta potential analysis was performed using a high-performance electrokinetic analyzer (SurPASS 3, Anton Paar, Austria). The streaming potential was measured between two meshes, each with dimensions of 20 × 10 mm, positioned in the cell with an adjustable gap set to 115 μm. The pH was controlled within the range of 5.5–9.0. The pH was controlled by gradually adding 0.05 M HCl or 0.05 M NaOH to a 0.01 M KCl solution. Zeta potential measurements were taken in four repetitions at each pH level, with the sample being replaced after each measurement. Results are presented as the average values, with error bars representing the standard deviation calculated from the four tests.

### 2.10 Cell culture studies

Human osteoblast-like cells (MG-63) (Sigma Aldrich, UK) were used for the cell culture study. Scaffolds were cut into 15 mm diameter circles, placed in 24-well plates, and sterilized under UV light for 30 min. Glass was used as a control. For each study, an equal cell density of 2 × 10^4^ cells per 1 ml of culture medium was used. Incubation took place at 37 °C with 95 % humidity in a 5 % CO_2_ atmosphere in an incubator (Memmert GmbH + Co. KG, Inc 108med, Schwabach, Germany). The cell culture medium consisted of Dulbecco's Modified Eagle Medium (DMEM with 4.5 g/L D-glucose, Biological Industries, Israel), supplemented with 10 % fetal bovine serum (FBS, Biological Industries, Israel), 2 % antibiotics (penicillin-streptomycin, Biological Industries, Israel), 1 % L-glutamine solution (Biological Industries, Israel), and 1 % non-essential amino acid solution (Sigma-Aldrich, USA).

### 2.11 Cell adhesion

Cells were seeded onto scaffolds for the cell adhesion evaluation and incubated for 5 h. After this time, samples were washed with phosphate-buffered saline (PBS, Biomed Lublin, Poland) to remove non-adherent cells. Subsequently, the scaffolds and control were fixed using 4 % paraformaldehyde for 15 min and then rinsed again with PBS. Nuclear staining was achieved by incubating the samples with 4′,6-diamidino-2-phenylindole (DAPI, Sigma-Aldrich, UK) for 15 min, followed by a final PBS rinse. The samples were investigated using a confocal laser scanning microscope (Zeiss LSM 900, Germany), and cell quantification was performed using 100 images per sample with CellProfiler 4.2.6 (Broad Institude, USA) software.

### 2.12 Confocal microscopy: nuclei, actin, focal adhesions

Cell actin was analyzed after 1, 3 and 7 days of incubation. Focal adhesions were visualized after 3 days. Staining protocols were similar to those previously reported [45]. Briefly, all immunofluorescence staining followed a procedure: scaffolds were fixed with 4 % paraformaldehyde (Sigma-Aldrich, UK) for 15 min, washed with PBS, and permeabilized with 0.1 % Triton X-100 (Sigma-Aldrich, UK) for 10 min. After blocking with 3 % BSA (Sigma-Aldrich, UK) in PBS for 60 min, focal adhesion complexes were stained with anti-paxillin antibodies (ab32084, Abcam, UK) overnight. Secondary staining with Alexa Fluor Plus 555 (A32732, Thermo Fisher, USA) and actin staining with Alexa Fluor 488 Phalloidin (Thermo Fisher, USA) were done for 1 h, followed by nuclear staining with DAPI (Sigma-Aldrich, UK) for 15 min.

Images were captured using a confocal microscope (Zeiss LSM 900, Germany) with objectives ranging from 10× to 63× . Excitation used laser lines at 405 nm, 488 nm, and 555 nm for DAPI, Alexa Fluor 488, and Alexa Fluor 555, respectively. Airyscan superresolution microscopy utilized a 63× oil objective with specific emission bands for fluorescence detection.

### 2.13 Confocal microscopy: cluster analysis

For each sample, images were captured in 15 different fields of view using both confocal and Airyscan modes. The confocal mode was used to capture transmitted light images of PLLA fibers, with visible Ti_3_C_2_T*_x_* and rGO agglomerates. The Airyscan mode was employed for high-resolution imaging of paxillin proteins, which are involved in cell adhesion processes. Confocal laser microscopy imaging parameters are presented in Table 2.

**Table 2:** The summary of all confocal laser microscopy imaging parameters.

Imaging mode	Objective	Laser wavelength	Detection wavelength	Pixel time	Pixel size
Confocal	Plan-apochromat 63x/1.4 Oil DIC M27	561 nm, 488 nm, and 405 nm for Alexa 555, Alexa 488, and DAPI, respectively	540–700 nm for Alexa 555, 450–545 nm for Alexa 488, and 400–600 nm for DAPI	1.91 μs	0.071 μm
Airyscan	Plan-apochromat 63x/1.4 Oil DIC M27	561 nm, 488 nm, and 405 nm for Alexa 555, Alexa 488, and DAPI, respectively	540–700 nm for Alexa 555, 450–545 nm for Alexa 488, and 400–600 nm for DAPI	3.82 μs	0.035 μm

The distribution of paxillin at cell adhesion sites on PLLA fibers (both with and without Ti_3_C_2_T*_x_* or rGO) was imaged and analyzed using ImageJ software (version 1.51, Fiji, USA). Fluorescence images were normalized using the Statistical Dominance Algorithm (SDA) to reduce noise and correct uneven illumination. Cell adhesion regions were manually defined, and the analysis focused on these areas. ImageJ tools were used to identify local maxima of signal intensity, and nearest-neighbor (NN) analysis was conducted to determine the minimum distances between the local peaks of paxillin and the identified Ti_3_C_2_T*_x_* or rGO agglomerations, following the previously established protocols [10].

Clustering of these points was analyzed using the Density-Based Spatial Clustering of Applications with Noise (DBSCAN), which identifies clusters and outliers based on the proximity of points. DBSCAN parameters (epsilon and minimum points) were optimized using a set of 10 images and then applied to the remaining data. Statistical analysis of the results was performed using OriginPro software (version 2022 9.9, USA).

### 2.14 Cell proliferation, replication and infiltration

Cell proliferation was measured using the CellTiter-Blue® Assay (GloMax Discover plate reader, Promega, USA) at 1, 3 and 7 days post-incubation. At each time point, the culture media was replaced with 1 ml of fresh media containing 20 % CellTiter-Blue® reagent (Promega, USA) and incubated for 4 h at 37 °C. After incubation, 100 μl of the reagent mixture was transferred from each well to a 96-well plate in triplicate, and fluorescence was recorded at 560/590 nm using the GloMax® Discover system (Promega, USA). Each type of scaffold and control glass was tested in duplicate. Statistical analysis of the data was carried out using OriginPro software (version 2022 9.9, USA).

For the replication assessment, cells grown on scaffolds and glass (as a control) were treated with 10 μM 5-ethynyl-2′-deoxyuridine (EdU) for 1 h after 1, 3 and 5 days of culture. Following treatment, the samples were washed with PBS, fixed with 4 % paraformaldehyde, and then permeabilized with 0.1 % Triton X-100 (Sigma-Aldrich, USA) and blocked in 3 % bovine serum albumin (Sigma-Aldrich, USA) at 25 °C. The incorporated EdU was detected using the ClickiT™ EdU AF488 imaging kit (Invitrogen/Molecular Probes, USA). Cells were stained with DAPI (Millipore, Germany) for 15 min. Fixed samples were imaged using a Zeiss LSM 900 confocal microscope (CLSM, Carl Zeiss Microscopy GmbH). For imaging, 405 nm and 488 nm laser lines were used for excitation, with emission detection bands of 410–500 nm for DAPI and 500–700 nm for Alexa Fluor™ 488 bound to the incorporated EdU. A Plan-Apochromat 20× /0.8 M27 objective was used for imaging. For each sample, over 60 images were captured from different fields of view. The images were recorded in two channels: the green channel, where the signal from Alexa 488-labeled EdU precursor incorporated into the cell was detected, and the blue channel, where the signal from labeled DNA in cell nuclei was captured. Nuclei detection in both channels was performed using the CellProfiler 4.2.6 software (Broad Institute, USA).

Cell infiltration into the scaffolds after 7 days of incubation was analyzed with CLSM. Parallel imaging of cell nuclei and actin was performed to provide a detailed evaluation of cellular distribution. All samples were fixed prior to imaging to preserve cellular morphology with DAPI and Alexa Fluor 488 Phalloidin. Imaging was conducted using Z-stacks, capturing the fluorescent signal. The imaging settings, including excitation light intensity, detection ranges, and detector gain, were kept consistent across all samples to ensure reliable and comparable results. Z-stacks were acquired with a step size of 1.7 μm. The acquisition range for each sample was individually adjusted, starting from the first in-focus layer where nuclei appeared and continuing deeper into the scaffold until the last visible labeled nuclei moved out of focus. From each layer within the Z-stack, the average pixel intensity was calculated. The depth corresponding to the maximum average pixel intensity in the Z-stack was identified as the plane with the highest density of in-focus nuclei, which was interpreted as the position of cells on the scaffold surface. In the resulting plots, the X axis represents the scanned depth for each sample, providing a visualization of cellular distribution and scaffold infiltration.

### 2.15 SEM of scaffolds with cells

SEM imaging of cells on PLLA scaffolds was carried out after 7 days of culture. The cells were fixed using 2.5 % glutaraldehyde (Sigma-Aldrich, UK) for 1 h at 25 °C. After fixation, the samples were rinsed three times with PBS and then dehydrated through a series of ethanol solutions (Avantor, Poland) with increasing concentrations (50 %, 60 %, 70 %, 80 %, 90 %, 100 %). The scaffolds were incubated in each ethanol solution for 7 min, repeating the process twice for the 100 % ethanol. Following dehydration, the samples were treated with hexamethyldisilazane (HMDS, Sigma-Aldrich, UK). Once the HMDS had fully evaporated, the samples were coated with an 8 nm layer of Au and imaged using the same SEM parameters as previously used for fiber characterization. Cell infiltration was assessed by visualizing cross-sections of scaffolds, which were cut through the middle of the sample using a scalpel and then coated with an Au layer using the protocol mentioned above.

## 3 Results and discussion

### 3.1 Scaffold's morphological, chemical, and mechanical characterization

Electrospinning is a straightforward technique for producing highly porous polymer scaffolds, with porosity exceeding 90 %, arranged in a random orientation. Thus, this method is widely used in tissue engineering, particularly for wound healing applications [46,47]. However, the interplay of the electric field and the surface tension in this electrohydrodynamic process is accompanied by many more parameters [48,49]. Importantly, whenever we start to create the hybrid scaffolds with rGO and MXene, one has to take into account the conductivity of the polymer solution. Following this essential line of thinking, we measured the conductivity for our three polymer solutions: PLLA, PLLA-rGO, and PLLA-Ti_3_C_2_T_*x*_ [48,50,51]. As expected, 2D conductive materials rGO and Ti_3_C_2_T_*x*_ enhanced the conductivity of the solution [[52], [53], [54]]. Results in Table 1 show that adding rGO increased the conductivity by approximately 11 μScm^−1^, while adding Ti_3_C_2_T_*x*_ increased it by 10 μScm^−1^. Consequently, due to the increased conductivity of solutions, the applied voltage and flow rate had to be adjusted to conduct electrospinning of fibers in a stable cone-jet mode (see Table 1). A similar observation was made based on experiments with polylactide (PLA) with the addition of cellulose nanocrystals [55].

**Fig. 2:**
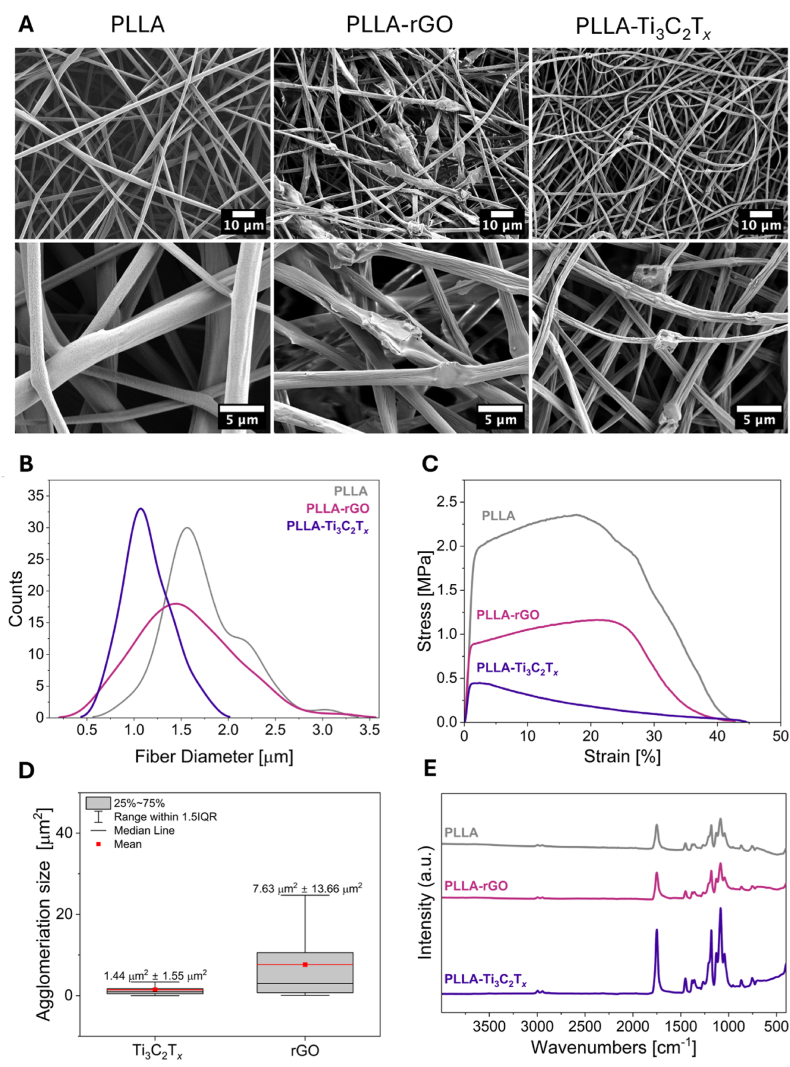
A) SEM micrographs of PLLA, PLLA-rGO, PLLA-Ti_3_C_2_T_*x*_ fibers. B) Fiber diameter distribution graph. C) Stress-strain curves from tensile tests of samples. D) Box chart of agglomeration size distribution within the fibers. E) FTIR results for fiber samples.

Fibers of PLLA-rGO and PLLA-Ti_3_C_2_T_*x*_ samples differ compared to PLLA in many aspects. Firstly, we analyzed the morphology of fibers using scanning electron microscopy (SEM), see Fig. 2A. Material agglomeration is present for both samples containing fillers and looks similar to beads, contrary to smooth and uniform PLLA fibers. The average diameter of fibers for all the samples is similar, as presented in the histogram (Fig. 2B). It is 1.74 ± 0.42 μm for PLLA fibers, while for fibers with fillers, the value slightly decreased, resulting in 1.56 ± 0.51 μm for PLLA-rGO and 1.14 ± 0.26 μm for PLLA-Ti_3_C_2_T_*x*_. It has been demonstrated previously that variations of ∼0.5 μm in fiber diameter do not significantly affect cell proliferation or differentiation when the diameters remain within the same order of magnitude [56]. Differences between the scaffolds are visible not only microscopically but also macroscopically, as presented in SI Figure S1 A, where it is shown that fillers affect the color of the scaffold. Moreover, to gather complex characterization of samples, we provide static contact angle measurements done with water, showing no significant differences, resulting in 117.6 ± 4.1°, 118.3 ± 2.9°, and 120.1 ± 4.5° for PLLA, PLLA-rGO, and PLLA-Ti_3_C_2_T_*x*_, respectively. Exemplary images of the water droplets on the scaffolds for this measurement are presented in SI Figure S1 B-D.

In terms of mechanical performance, the agglomeration of Ti_3_C_2_T_*x*_ and rGO within PLLA fibers significantly impacts their tensile properties, as shown in stress-strain curves (Fig. 2C). Fig. 2C presents the average curves from 5 measurements per sample, while all the curves are presented in SI Figure S1 E-G. Table 3 summarizes mechanical properties, highlighting evident differences between all samples. The Young's modulus of randomly oriented electrospun fiber mats was not determined due to fiber slippage and non-linear deformation behavior during tensile testing. As already been indicated in several studies the obtained values of the Young's modulus from not aligned electrospun fibers mats can be misleading [[57], [58], [59]]. Pure PLLA fibers exhibit good mechanical properties, with an elongation at break (Ɛ_f_) of 42 ± 5.29 %, a tensile strength (σ) of 2.41 ± 0.18 MPa, strain at maximum stress (Ɛ_max_) of 21.86 ± 4.56 %, and a toughness (W) of 70.62 ± 15.26 MJ m^−3^. The tensile test results for PLLA fibers are comparable to those reported in the literature for electrospun mats made of PLA and other polymers commonly used in tissue engineering [55,60,61]. Incorporating rGO into PLLA fibers results in a slight increase in Ɛ_f_ to 43.43 ± 1.85 %, indicating that rGO has a minor effect on the flexibility of the composite. However, compared to PLLA fibers, the tensile strength of PLLA-rGO fibers decreases significantly to 1.08 ± 0.19 MPa, while toughness drops to 30.37 ± 5.40 MJ m^−3^. The large agglomeration sizes observed for rGO, contribute to this reduction in strength and toughness. These regions of high-stress act as initiation sites for crack propagation, diminishing the overall mechanical integrity of the fibers [62]. Moreover, PLLA-Ti_3_C_2_T_*x*_ composite fibers exhibit an even more significant decrease in mechanical performance compared to PLLA and PLLA-rGO samples, with a lower tensile strength of 0.45 ± 0.10 MPa and a drastic reduction in Ɛ_max_ to 2.17 ± 0.50 %. The toughness is also reduced to 8.34 ± 1.43 MJ m^−3^, reflecting a substantial drop in the deformation energy the composite can absorb before failure.

**Table 3:** Tensile test results with the characteristics values of elongation at failure (Ɛ_f_), tensile strength (σ), toughness (W), and elongation at maximum stress (Ɛ_max_). Errors are based on standard deviation, with N = 5, where N is the tensile test of one mat sample.

Sample	Ɛ_f_ [%]	σ [MPa]	Ɛ_max_ [%]	W [MJ·m^−3^]
PLLA	42 ± 5.29	2.41 ± 0.18	21.86 ± 4.56	70.62 ± 15.26
PLLA-rGO	43.43 ± 1.85	1.08 ± 0.19	20.76 ± 0.65	30.37 ± 5.40
PLLA-Ti_3_C_2_T_*x*_	45.16 ± 3.29	0.45 ± 0.10	2.17 ± 0.50	8.34 ± 1.43

To investigate the role of filler agglomeration size and distribution on the fiber properties, we analyzed agglomeration patterns of Ti_3_C_2_T_*x*_ and rGO within the fiber structures using confocal laser scanning microscopy (CLSM) and its transmitted light imaging. Consistent imaging parameters were applied across all samples, capturing 20 images per sample to ensure a comprehensive agglomerate distribution and size assessment. Agglomerations of Ti_3_C_2_T_*x*_ and rGO were identified and analyzed within the fiber regions (SI Figure S1 H, J), delineated by the red mask (SI Figure S1 I, K).

Quantitative analysis revealed distinct differences in agglomeration size between the two fillers, as illustrated in the box plot in Fig. 2D. The average agglomeration size of rGO was significantly larger (7.63 ± 13.66 μm^2^) compared to Ti_3_C_2_T_*x*_ (1.44 ± 1.55 μm^2^). rGO also exhibited greater variability, as indicated by a broader interquartile range and overall range in the box plot. In contrast, Ti_3_C_2_T_*x*_ showed a narrower distribution and smaller agglomerates than rGO, suggesting better dispersion within the polymer matrix. These differences can be connected to distinct solvent-nanosheet interactions observed for Ti_3_C_2_T_*x*_ and rGO when dispersed in DMF. Ti_3_C_2_T_*x*_ is naturally terminated with surface functional groups (-OH, -F, -O) after the selective etching [63]. The presence of these polar functional groups renders its dispersion in polar solvents like DMF, as the polarity index of the solvent closely matches with the polarity of the material [64]. Here, the Hildebrand and Hansen models can be employed to predict the dispersion stability of nanomaterials in solvents. The Hildebrand solubility parameter (δ_T_) is defined as the square root of the cohesive energy density, whereas the Hansen parameters refer to the contribution from dispersion, dipole, and hydrogen bonding interactions. According to these models, Ti_3_C_2_T_*x*_ is stable in solvents with a δ_T_ close to 25.1 MPA^1/2^ [65], which aligns well with DMF (δ_T_ of 24.9 MPA^1/2^). Furthermore, the Hansen model describes that Ti_3_C_2_T_*x*_ is stable in solvents with high polarity and high dispersion interaction strength, explaining their favorable dispersion in polar solvents [64]. In contrast, the reduction process of rGO removes oxygen-containing surface functional groups, leading to a loss of surface polarity. Consequently, rGO exhibits poor dispersibility in DMF compared to Ti_3_C_2_T_*x*_. The δ_T_ of rGO is approximately 22 MPA^1/2^ [66], but this value varies depending on the degree of reduction, indicating a lower compatibility with DMF.

Additionally, by energy-dispersive X-ray spectroscopy (EDS) we confirmed the presence of Ti_3_C_2_T_*x*_ in the fibers, as shown by elemental mapping (SI Figure S1 L-N). Channels highlighting carbon (C), titanium (Ti), and fluorine (F) (SI Figure S1 N), as well as isolated Ti and F maps, verified the material's integration into fibers, appearing as agglomerates in beads-like features (SI Figure S1 L,M). The EDS spectrum for the PLLA-Ti_3_C_2_T_*x*_ sample (SI Figure S1 O) confirmed the presence of Ti. In summary, both fillers influence fiber mechanical properties, with their agglomeration size and distribution playing a critical role. Despite the smaller and more uniform agglomerates of Ti_3_C_2_T*_x_*, its presence in the PLLA matrix contributes to greater deterioration of tensile properties compared to rGO. This highlights the need to carefully consider filler dispersion and agglomeration characteristics in the design of composite materials.

As a necessary verification step, we performed Fourier-transform infrared spectroscopy (FTIR), see Fig. 2E. In the FTIR spectra of the composite fibers, characteristic peaks corresponding to the PLLA matrix were clearly visible. Although vibrations associated with Ti_3_C_2_T_*x*_ —such as Ti–C stretching—are typically reported around 469 cm^−1^, this peak was not clearly distinguishable in our samples. This is likely due to overlapping signals from the polymer and the relatively low filler content. As a result, identifying MXene-specific peaks using FTIR is challenging in this system, particularly in the 400–800 cm^−1^ region where such features are expected to appear [39,67]. Therefore, we acknowledge the limitations of FTIR in confirming the presence of MXenes in our composites and rely on additional characterization techniques (e.g., EDS, confocal imaging) to confirm their incorporation. The analysis of Ti_3_C_2_T_*x*_ powder is presented in SI Figure S1 P. Characteristic peaks of rGO could not also be distinguished in PLLA-rGO fibers due to the overlapping of peaks with those coming from PLLA material. Analysis of pure rGO powder revealed increased intensity at 1715 cm^−1^ assigned to the stretching vibration of C=O moiety, and 1634 cm^−1^, which can be attributed to the absorbed water molecules (SI Figure S1 P) [68]. The FTIR spectrum of all three samples of fibers reveals several distinctive peaks associated with PLLA chemical structure. A prominent peak at 1750 cm^−1^ corresponds to the C=O stretching mode of the ester group [69]. Peaks at 1211 and 1182 cm^−1^ are attributed to the C–O–C and CH_3_ groups, respectively. Other characteristic bands appear at 1044, 1087, 1132, and 1456 cm^−1^, linked to the asymmetric deformation of the C-CH_3_, C–O–C, C-CH_3_, and CH_3_ groups, respectively [70]. Stretching vibrations of the CH_3_ group are further indicated by peaks at 2946 and 2996 cm^−1^ [71]. The peak at 1384 cm^−1^ is also assigned to C–H stretching. Weaker bands at 872 and 921 cm^−1^ suggest the presence of α-crystal, while the band at 755 cm^−1^ is associated with C=O bending [71,72].

To complement the chemical verification provided by FTIR, differential scanning calorimetry (DSC) was performed to assess the thermal properties of the composite fibers and evaluate whether the incorporation of Ti_3_C_2_T_*x*_ and rGO affected the thermal behavior of the fibers. The DSC analysis (SI Figure S1 R) revealed no significant variation in the glass transition temperature (*T_g_*) among the samples. The measured *T_g_* values were 60.44 ± 0.51 °C for neat PLLA, 60.02 ± 0.22 °C for PLLA-rGO, and 60.27 ± 0.59 °C for PLLA-Ti_3_C_2_T_*x*_. These results indicate that the addition of Ti_3_C_2_T_*x*_ and rGO did not alter the crystalline structure and further the thermal stability of the polymer matrix [73]. The consistency in *T_g_* across all samples suggests limited interaction between the fillers and the polymer chains at a level that would significantly influence the thermal transitions. Comparing these findings with the literature is difficult because changes in the polymer structure are dependent on the composite fabrication process. However, similar results were reported with no change in *T_g_* caused by incorporating rGO in polycaprolactone (PCL) or PLA matrix [73,74]. Although our thermal analysis did not reveal strong interactions between the polymer and fillers, previous studies suggest that conductive fillers within an insulating polymer matrix can still enhance charge transport, influencing the properties of composite fibers. For example, Wang et al. demonstrated that in polyethylene/graphene nanocomposites, improved conductivity was achieved through trap-modulated charge carrier transport, even with minimal polymer–filler interaction [75].

### 3.2 Surface properties of scaffolds

XPS analysis was performed to verify the surface chemistry of electrospun fibers. Table 4 lists the surface concentrations of chemical bonds obtained from fitting XPS data for all analyzed samples. The C 1s spectra were fitted with three components typical for PLLA polymer [76]. The first line centered at 285.0 eV indicates the presence of aliphatic carbon C-C, the second line at 286.9 eV indicates the presence of C-O groups, and the third line at 289.0 eV comes from O-C=O type bonds [76]. The O 1s spectra were fitted with three components: the first line centered at 532.2 eV, which points out the existence of O=C type bonds; the second line centered at 533.6 eV, indicating the presence of O-C type bonds, both typical for PLLA polymer and the last line centered at 535.0 eV comes from the presence of adsorbed water [76,77]. There is also a small amount of silicon in both metallic state and oxide form (from the substrate, which is a silicon wafer). High-resolution spectra are presented in SI Figure S2. The XPS analysis reveals that the surface chemistry of all samples is characterized only by bonds typical for PLLA, not the rGO and Ti_3_C_2_T_*x*_. It suggests that most of the fillers are blended inside the fibers during electrospinning, as the XPS analysis depth is up to 5.7 nm from the fiber surface at a 45° measurement angle [78,79]. If the filler mixed with the PLLA solution were located at the surface, the XPS analysis would be able to detect that [80]. Similar surface chemistry of all the samples let us exclude this parameter from the possible effect on cell response.

**Table 4:** Atomic composition (%) of the surface of electrospun samples determined by fitting XPS data, see SI Figure S2.

	C			O			Si
Binding energy [eV]	285.0	286.9	289.0	532.2	533.6	535.0	100–103
Group	C-C	C-O	O-C=O	O=C	O-C O-Si	H_2_O_ads_	Si SiO_x_
PLLA	22.5	16.7	23.9	16.4	17.0	2.6	0.9
PLLA-rGO	21.0	18.2	20.8	17.3	17.7	2.7	2.3
PLLA-Ti_3_C_2_T_*x*_	18.1	16.4	21.6	15.0	17.5	4.4	6.9

**Fig. 3:**
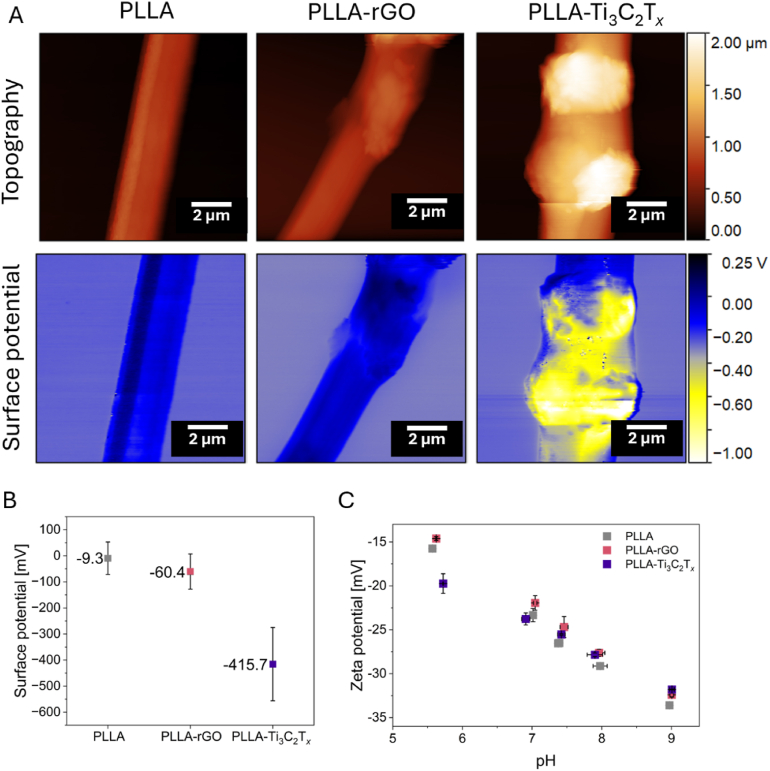
Surface properties of fibers: A) topography images and surface potential maps (KPFM), B) surface potential of fibers (KPFM), and C) zeta potential results in the pH range of approximately 5.5–9.

As discussed in the literature, the changes in the surface chemistry of electrospun fibers can be correlated with the surface potential variation measured directly by KPFM or validated by zeta potential investigation, which highly affects cell integration with the scaffolds [81,82]. For instance, it was reported that the charges on the fibers can be tuned by changing voltage polarity during electrospinning, further improving cell adhesion [45,83]. Fig. 3A shows the KPFM maps illustrating the fiber topography and the corresponding surface potential. The KPFM measurements revealed that fibers with rGO exhibited an average surface potential shift of approximately 50 mV compared to pure PLLA fibers, as presented in Fig. 3B. Notably, fibers containing Ti_3_C_2_T_*x*_ displayed an even greater surface potential shift, exceeding 400 mV relative to pure PLLA. The observed change in surface potential is likely due to a shift in the material's work function after blending PLLA with rGO and Ti_3_C_2_T_*x*_ fillers. It is due to the relationship between work function and the contact potential difference obtained in the KPFM measurement as stated in the equation:

(1) $$V_{\text{CPD}}=\frac{{\text{WF}}_{\text{tip}}-{\text{WF}}_{\text{sample}}}{e}$$

where ${\text{WF}}_{\text{tip}}$ and ${\text{WF}}_{\text{sample}}$ are the work functions of the tip and the sample, respectively, and *e* is the elementary charge [84]. In this work, we determined the work function (WF) of PLLA using highly ordered pyrolytic graphite (HOPG) as a reference, obtaining a value of 4.7 ± 0.1 eV. This estimated WF is consistent with previous findings, where, although the WF of PLLA was not directly reported, data suggested a similar range, allowing us to approximate a WF of 4.6 ± 0.1 eV [45]. Therefore, we estimate the WF of electrospun PLLA to be in the range of 4.6–4.7 eV. Thus, the relatively small shift in surface potential for the PLLA-rGO fibers is probably due to the similarity between the WF of PLLA and rGO, which is approximately 4.8 eV according to the literature, which results in minimal impact on the overall surface potential of PLLA-rGO fibers [85]. In contrast, PLLA-Ti_3_C_2_T_*x*_ fibers showed a substantial increase in surface potential, likely due to the lower WF of Ti_3_C_2_T_*x*_ (∼4.2 eV) [86] The introduction of Ti_3_C_2_T_*x*_ filler thus shifted the WF of the composite material, resulting in the observed change in surface potential. It is important to note that these measurements were conducted under ambient conditions, which may introduce variations and contribute to the observed standard deviation in WF calculations. While KPFM provides localized surface potential measurements on individual fibers, the consistent trends observed across multiple sites suggest these values are representative. The data demonstrate a clear correlation between the filler type and the resulting surface potential, highlighting the potential of rGO and MXene for tuning fiber surface properties in relevant applications.

Results given by XPS and KPFM prove that even if the incorporated particles are not exposed on the surface being covered with a layer of polymer thicker than 6 nm, they still affect the surface charge of fibers. As KPFM measures the local surface potential at the nanoscale, providing the data on the work function or contact potential difference, this is a direct reflection of surface charge density, but on a dry, solid surface in a controlled environment. In contrast, zeta potential indicates the electrostatic potential near the slipping plane in a liquid environment, reflecting the charge that can develop when the material is suspended in a solution [87]. We performed zeta potential measurements to give a more comprehensive view of the material behavior across different conditions (Fig. 3C). The results show no significant differences between the samples in zeta potential values in the pH range of 5.5–9. We have chosen this pH range to reflect conditions relevant to the human body, encompassing the physiological pH spectrum commonly observed in various biological systems. For pH around 7.5, zeta potential was −26.61 mV for PLLA, −24.7 mV for PLLA-rGO, and −25.52 mV for PLLA-Ti_3_C_2_T_*x*_. Work published by Yin and Drelich discusses the role of electrokinetic effects and surface heterogeneity challenges in zeta potential measurements [88]. Their study highlights how zeta potential measurements can often average out the actual heterogeneity of surface charges, as traditional electrokinetic approaches do not provide localized information and only offer an overall potential across complex surfaces. Due to the complex composition of the cell culture media, the interpretation of the zeta potential data is challenging, however, it has been previously validated for the simulated body fluid [89].

### 3.3 Cell adhesion and focal adhesion point analysis

**Fig. 4:**
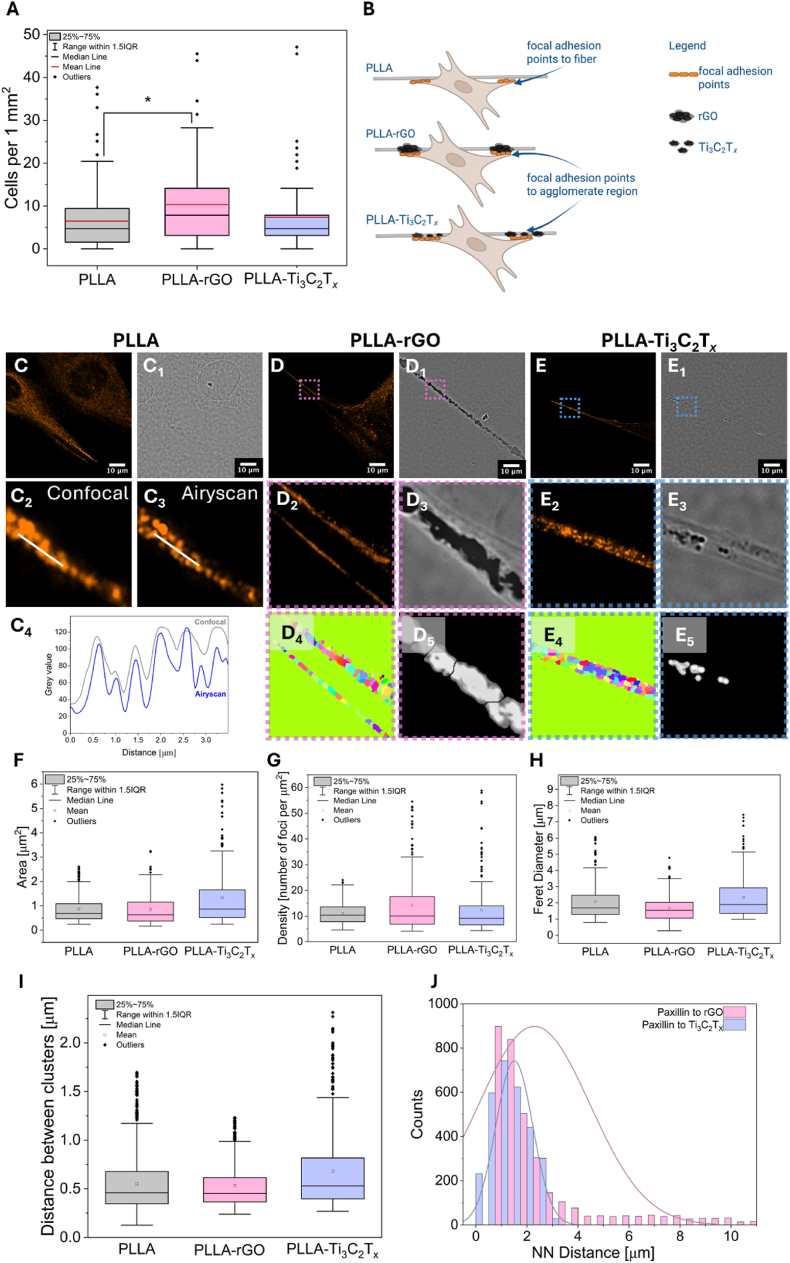
A) Cell adhesion after 5 h of incubation. B) Scheme presenting the focal adhesion point analysis of cells to PLLA fibers, and fibers with filler enriched regions (rGO, and Ti_3_C_2_T_*x*_), underlying single fiber – single cell interaction. High-resolution imaging and quantitative analysis of cellular interaction with different scaffolds: C) CLSM image of single cell and single PLLA fiber with stained paxillin (orange), and C_1_) the same view in transmitted light. C_2_), C_3_) Graph displaying the differences in the resolution contrast between CLSM and high-resolution CLSM (Airyscan mode) imaging. C_4_) Graph presenting the intensity profile, which demonstrates improved spatial resolution of Airyscan mode compared to confocal imaging. D) CLSM image of single cell and single PLLA-rGO fiber with stained paxillin (orange), and D_1_) the same view in transmitted light. D_2_), D_3_) Zoomed images on selected areas from D) and D_1_) marked with pink squares. D_4_), D_5_) Segmented paxillin and rGO agglomerations for quantitative analysis, respectively. E) CLSM image of single cell and single PLLA-Ti_3_C_2_T_*x*_ fiber with stained paxillin (orange), and E_1_) the same view in transmitted light. E_2_), E_3_) Zoomed images on selected areas from E) and E_1_) marked with blue squares. E_4_), E_5_) Segmented paxillin and Ti_3_C_2_T_*x*_ agglomerations for quantitative analysis, respectively. F) Box chart presenting cluster analysis of the area of focal adhesions. G) Graph showing foci density, and H) Feret diameter of focal adhesions. I) Box chart presenting the distance between the clusters, and J) graph of nearest-neighbor (NN) distance distribution between rGO and Ti_3_C_2_T_*x*_ agglomerations and focal adhesions. Focal adhesions (paxillin) stained with Alexa Fluor Plus 555 (orange). (For interpretation of the references to color in this figure legend, the reader is referred to the Web version of this article.)

Cell adhesion after 5h of incubation, followed by scaffold rinsing to remove non-adhered cells, was measured as cell density per mm^2^ of fibrous scaffold (see Fig. 4A). Here, PLLA and PLLA-Ti_3_C_2_T_*x*_ demonstrated similar adhesion of osteoblasts. In contrast, PLLA-rGO showed significantly higher cell adhesion than pure PLLA fibers, reflected by the greater median cell density and a wider range of values, suggesting strong cell-fiber interactions. This suggests that rGO can enhance physical adhesion due to rGO agglomerates being much bigger in size (SI Figure S1 H-K), changing scaffold geometry, and creating supportive architecture. To the best of our knowledge, agglomerated fillers were never used to diversify the architecture of electrospun fibers for better cell-material interaction in tissue engineering. Interestingly, beaded electrospun fibers have similar morphology and have recently drawn attention in drug delivery systems [[90], [91], [92]]. A study by Santillan et al. presents that beads-on-string fibers benefit osteoblast attachment and filipodia formation, serving more attachment sites [93].

Since adhesion after 5 h is an initial cell response, we additionally provide a detailed analysis of focal adhesion points after 3 days of incubation. Here, we use the protocol of osteoblast's focal adhesion formation assessment that was already published for electrospun PMMA fibers [10]. Similarly, we verified cell-individual fiber interaction as presented in the scheme in Fig. 4B. In this study, paxillin, a key protein forming the multiprotein complex responsible for cell adhesion to the extracellular matrix, was labeled using immunofluorescence. Paxillin, visualized in orange in the images, enabled detailed analysis of adhesion sites. High-resolution CLSM (Airyscan) imaging provided high-quality images of paxillin distribution within cells, subsequently used for quantitative analysis of cell adhesion site distribution on different scaffolds. In Fig. 4: C, C_1_, D, D_1_, E, E_1_ we present CLSM images of focal adhesions (stained paxillin - orange) and transmitted light channel for single fiber and single cell for PLLA, PLLA-rGO, and PLLA-Ti_3_C_2_T_*x*_ fibers. The application of high-resolution CLSM imaging enabled precise visualization of the distribution of cell adhesion sites to the scaffold fibers (C_2_, C_3_, C_4_). High-resolution CLSM imaging resulted in a higher signal-to-noise ratio, allowing for the identification of a greater number of distinguishable adhesion sites and structural features, which were not as clearly resolved with confocal imaging [10,94,95]. Agglomerations of rGO and Ti_3_C_2_T_*x*_ are visibly seen in transmitted light Fig. 4: D_1_, D_3_, E_1_, E_3_, respectively.

The quantitative analysis involved binary processing and quantification of the labeled adhesion site signals, followed by cluster and morphological analyses. The cluster analysis, see Fig. 4: D_4_, E_4_, utilized the DBSCAN algorithm to identify and quantify the distribution of adhesion sites. Morphological analysis focused on determining fundamental parameters, including: area (the size of identified adhesion clusters, see Fig. 4F), density (the number of detected intensity maxima within each cluster area see Fig. 4G), Feret diameter (the maximum dimension of the adhesion clusters, see Fig. 4H), distance between clusters (the spatial arrangement and separation between adhesion clusters, see Fig. 4I). For regions where cells interacted with Ti_3_C_2_T_*x*_ or rGO containing fibers, additional analysis was conducted. This involved measuring the distance from each detected local intensity maximum (adhesion site) to the centroid of the corresponding Ti_3_C_2_T_*x*_ or rGO area. These measurements provided detailed insights into how scaffold properties influence the spatial organization of adhesion sites (Fig. 4J) [10].

The analysis of the area of cell adhesion clusters (Fig. 4F) revealed notable differences depending on the scaffold material. The average cluster size for cell adhesion to PLLA fibers was comparable to that observed for PLLA-rGO fibers, indicating similar interactions between cells and these scaffolds in terms of adhesion area. However, in the case of cells adhering to PLLA-Ti_3_C_2_T_*x*_ fibers, the average cluster size was over 50 % larger than those observed for the other two materials. This significant increase suggests that the inclusion of Ti_3_C_2_T_*x*_ in the scaffold composition enhances the development of larger adhesion sites. This could be attributed to the unique surface potential of PLLA-Ti_3_C_2_T_*x*_ fibers, which may promote stronger or more extensive interactions between cells and the scaffold surface.

The density (Fig. 4G), representing the local maxima per millimeter within identified adhesion clusters, did not reveal significant differences between the tested scaffolds. However, a wider spread of values was observed for adhesion to PLLA-Ti_3_C_2_T_*x*_ and PLLA-rGO fibers. For adhesion complexes on PLLA fibers, the variability in this parameter was approximately two times lower, suggesting a more uniform cell adhesion process. This can be explained by the cells interacting either directly with regions enriched with Ti_3_C_2_T_*x*_ or rGO agglomerates or with areas of the scaffold lacking these additives.

The Feret diameter (Fig. 4H), defined as the maximum distance between two points on the outer contour of an object along a specific direction, provides insights into the elongation of adhesion clusters on different scaffolds. The results indicate that the most elongated adhesion clusters were observed in the interactions between cells and PLLA-Ti_3_C_2_T_*x*_ fibers, with an average Feret diameter of 2.35 μm. Additionally, this scaffold type exhibited the highest variability in Feret diameter distribution among all tested scaffolds, suggesting diverse morphologies of adhesion clusters. In contrast, the least elongated adhesion clusters were associated with cell adhesion to PLLA-rGO fibers, with an average Feret diameter of 1.68 μm and the smallest variability in distribution. This observation implies that adhesion clusters on PLLA-rGO fibers are more compact and uniform in shape than those on PLLA-Ti_3_C_2_T_*x*_ or pure PLLA fibers.

The analysis of the distance between clusters (Fig. 4I) examined the spatial distribution of adhesion clusters on different scaffold types. The results revealed no statistically significant differences in the average distance between adhesion clusters on PLLA and PLLA-rGO fibers, indicating a similar spatial arrangement of adhesion sites on these materials. In contrast, interactions between cells and PLLA-Ti_3_C_2_T_*x*_ fibers showed a broader spread in the measured distances between clusters. Additionally, the average distance between adhesion clusters on PLLA-Ti_3_C_2_T_*x*_ was approximately 30 % greater than that observed for PLLA and PLLA-rGO fibers. This increased variability and larger average distance suggest that the incorporation of Ti_3_C_2_T_X_ alters the spatial organization of adhesion clusters, potentially due to the unique surface properties or very good distribution of Ti_3_C_2_T_*x*_ within the scaffold.

The histogram in Fig. 4J represents the distribution of measured distances between the center of intensity for each identified paxillin accumulation site (as a marker of cell adhesion) and the centroid of polymer fibers containing either Ti_3_C_2_T_*x*_ or rGO. This analysis provides insight into how cells form adhesion sites relative to regions enriched with Ti_3_C_2_T_*x*_ or rGO in PLLA fibers. The results presented in this histogram (Fig. 4J) indicate that for PLLA-rGO fibers, cells exhibit a stronger tendency to form adhesion sites in close proximity to or directly at fiber regions rich in rGO, compared to Ti_3_C_2_T_*x*_. In both cases, the majority of identified adhesion sites are located near regions containing Ti_3_C_2_T_*x*_ or rGO. However, a significant portion of adhesion sites associated with PLLA-Ti_3_C_2_T_*x*_ fibers are found at greater distances from the "Ti_3_C_2_T_*x*_-enriched" regions. This observation is somewhat counterintuitive. Ti_3_C_2_T_*x*_-rich regions in fibers are smaller and more uniformly distributed compared to rGO-rich regions, theoretically giving cells more opportunities to form adhesion sites close to Ti_3_C_2_T_*x*_ agglomerates. Conversely, rGO clusters within the fibers are significantly larger, making it expected that the measured distances between adhesion sites and the centroids of rGO-enriched regions would be greater, but surprisingly, this is not the case. This phenomenon can be explained by the differences in the accessibility and spatial distribution of rGO and Ti_3_C_2_T_*x*_ within the PLLA fibers. Data from Fig. 2D and SI Figure S1 H-K highlight substantial differences in the distribution of Ti_3_C_2_T_*x*_ and rGO within the fibers. Cells have relatively greater access to rGO-enriched regions within PLLA-rGO fibers, which encourages the formation of adhesion sites directly at these regions. In contrast, the smaller and more evenly dispersed Ti_3_C_2_T_*x*_ agglomerates result in cells forming adhesion sites at varying distances from Ti_3_C_2_T_*x*_ regions. The larger rGO agglomerates within fibers promote more consistent and closer adhesion, whereas the smaller, more uniform Ti_3_C_2_T_*x*_ areas lead to a broader distribution of adhesion distances.

Summarizing, cluster and morphological analyses revealed significant differences in the architecture of cell adhesion sites formed on PLLA fibers compared to PLLA fibers with Ti_3_C_2_T_*x*_ or rGO additives. Cell adhesion clusters on PLLA-Ti_3_C_2_T_*x*_ fibers were observed to be longer, larger, and denser in terms of local maxima of adhesion sites compared to those on the other fiber types. This indicates that the addition of Ti_3_C_2_T_*x*_ significantly alters the adhesion site architecture, promoting the formation of more extensive and dense adhesion clusters. In contrast, the adhesion clusters formed on PLLA and PLLA-rGO fibers exhibited similar morphological characteristics, including comparable sizes and elongation. However, cells interacting with PLLA-rGO fibers formed a denser network of local adhesion sites compared to those on pure PLLA. It is important to note that the initial cell adhesion measured after 5 h reflects early attachment dynamics, whereas the analysis of focal adhesion points via paxillin staining after 3 days provides insight into more mature and stabilized cell–material interactions, which follow different trends over time.

### 3.4 Cell proliferation, replication, and infiltration into the scaffold

**Fig. 5:**
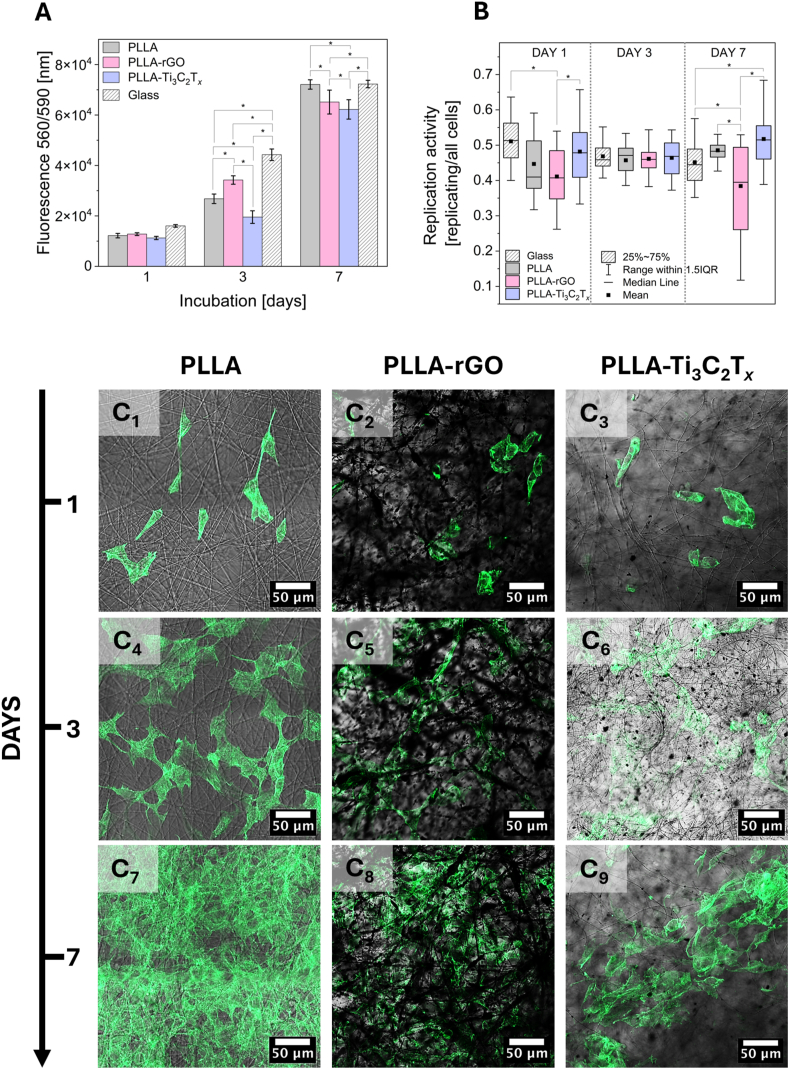
A) Proliferation and B) replication assessment after 1, 3, and 7 days of cell incubation on three types of scaffolds and glass slide as a positive control. Morphology of cells on PLLA, PLLA-rGO, and PLLA-Ti_3_C_2_T_*x*_ fibers at 1 (C_1_-C_3_), 3 (C_4_-C_6_), and 7 (C_7_-C_9_) days of culture visualized with CLSM. The actin filaments were stained with Alexa Fluor 488 Phalloidin (green). ∗statistical significance calculated with ANOVA followed by Tukey's post hock tests, p < 0.05. (For interpretation of the references to color in this figure legend, the reader is referred to the Web version of this article.)

Cell proliferation is the critical step of material biocompatibility assessment, which gives information on how well cells grow and multiply on the material, indicating the material's ability to support cellular functions vital for tissue regeneration and repair [[96], [97], [98], [99], [100], [101]]. The surface properties of electrospun fibers, such as topography, porosity, architecture, surface chemistry, and surface charges, play a role in influencing cell proliferation by modulating cell adhesion, nutrient diffusion, and signaling pathways [21,45]. Hence, proliferation, replication, and adhesion provide complementary evaluations of cell behavior in response to fiber surface properties [12,[102], [103], [104]]. The replication test is used to detect new DNA synthesis during cell replication. This assay enables the measurement of DNA synthesis activity, accurately determining whether cells are undergoing division during the specific time window in which they were incubated with the precursor [105]. It offers high sensitivity and specificity, helping in the interpretation of the results of standard proliferation and adding information about its dynamics [102,[106], [107], [108], [109]]. Fig. 5A presents results from the proliferation of cells on different substrates: PLLA, PLLA-rGO, and PLLA-Ti_3_C_2_T_*x*_ fibers at days 1, 3, and 7 of incubation. On day 1, all materials showed similar cell proliferation, suggesting no significant difference in initial compatibility. Day 3 results indicate significantly the best proliferation for PLLA-rGO, slightly lower for PLLA, and lastly for PLLA-Ti_3_C_2_T_*x*_. The last time point at day 7 presents significantly increased proliferation for all of the samples, with the highest fluorescence coming from PLLA fibers, followed by PLLA-rGO and PLLA-Ti_3_C_2_T_*x*_. Replication activity, defined as the ratio of replicating to all cells, was relatively similar for all samples on days 1 and 3, see Fig. 5E. In contrast, at day 7, PLLA-Ti_3_C_2_T_*x*_ displayed the highest replication activity, significantly higher than for PLLA-rGO. Exemplary CLSM images used for the replication study are shown in SI Figure S3, where green nuclei represent the replicating and blue non-replicating cells.

The confocal images in Fig. 5C_1_-C_9_ demonstrate the increased number of cells on all scaffolds over time, starting from day 1 (Fig. 5C_1_-C_3_), progressing through day 3 (Fig. 5C_4_-C_6_), and culminating at day 7 (Fig. 5C_7_-C_9_). During this period, the cell morphology remains consistent with standard cellular structures, indicating no adverse effects on cell morphology. By day 7, a monolayer of cells is evident on the surface of the PLLA fibers. In contrast, for the PLLA-rGO and PLLA-Ti_3_C_2_T_*x*_ scaffolds, the cells appear less densely packed on the top surface of fibers.

**Fig. 6:**
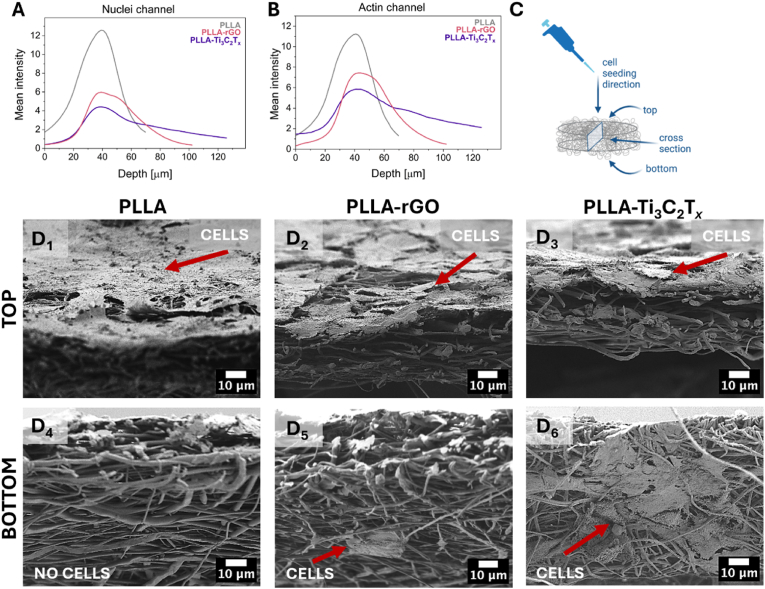
Analysis of signal intensity from immunofluorescently stained actin and nuclei of osteoblasts on varying depths of z-stack imaging of PLLA, PLLA-rGO, and PLLA-Ti_3_C_2_T_*x*_ scaffolds (7th day of incubation), with depth 0 μm being the top view of the sample. A) Graph with the signal intensity of nuclei channel (DAPI), and B) the actin channel (Alexa Fluor 488 Phalloidin). C) Scheme of cell seeding with indicated top and bottom view of sample observation. D_1_-D_6_) SEM micrographs of cells after 7 days of culture on PLLA, PLLA-rGO, and PLLA-Ti_3_C_2_T_*x*_ fibers, taken at: D_1_-D_3_) top view, D_4_-D_6_) bottom view of the samples, allowing analysis of cell infiltration into the scaffolds.

Due to observed differences in the properties of scaffolds and varieties in cell responses, we provide a study on cell infiltration into the fibrous structure. Cells are visibly growing into the scaffold structure, as evidenced by cell bodies extending out of the focal plane of the confocal images (Fig. 5C_7_-C_9_). This observation was further confirmed by 3D CLSM imaging (z-stack) and signal depth analysis, which captured signals from both actin filaments and nuclei presented in a digital movie (SI Movie S1). This technique confirms cellular penetration throughout the PLLA, PLLA-rGO and PLLA-Ti_3_C_2_T_*x*_ scaffolds, and CLSM signal depth analysis gives insights into cell infiltration. The mean intensity profiles of nuclei (Fig. 6A) and actin (Fig. 6B) across the depth of the scaffolds PLLA, PLLA-rGO, and PLLA-Ti_3_C_2_T_*x*_ highlight the variations in cellular behavior. The actin signal for PLLA scaffolds peaks with a full width at half maximum (FWHM) of 30.1 μm. This sharp and narrow peak indicates that cells predominantly reside near the scaffold's surface, with very limited migration into deeper layers. Similarly, the nuclei signal shows a maximum intensity of 10.9 with an FWHM of 29.7 μm, confirming restricted cellular penetration. The PLLA-rGO scaffold exhibits a broader actin signal profile, with a maximum intensity of 5.8 and an FWHM of 52.1 μm. This indicates enhanced cellular infiltration throughout the scaffold, with cells distributed over a wider depth range, reaching depths up to 100 μm. The nuclei signal also supports this observation, with a maximum intensity of 4.0 and an FWHM of 48.3 μm. The broader depth distribution in PLLA-rGO sample (up to ∼100 μm) reflects improved scaffold properties, which promote cellular migration and integration compared to PLLA. The actin signal for the PLLA-Ti_3_C_2_T_*x*_ scaffold shows a maximum intensity of 7.4 μm and an FWHM of 37.3 μm. This distribution suggests the best cellular infiltration, with cells extending deeper into the scaffold than for PLLA and PLLA-rGO. The cells penetrate to depths of approximately 120 μm. The nuclei signal mirrors this trend, with a maximum intensity of 5.6 μm and an FWHM of 38.0 μm. This suggests that PLLA-Ti_3_C_2_T_*x*_ scaffolds provide the most preferable environment for cellular migration into deeper layers (up to ∼120 μm), while the PLLA scaffold limits cellular penetration to surface layers, with cells concentrated within the first ∼30 μm from the surface.

Supplementary data related to this article can be found online at https://doi.org/10.1016/j.mtbio.2025.101785

The following are the Supplementary data related to this article.

Video 1 Video 1

The scheme in Fig. 6C shows the methodology of seeding cells and choosing the plane for top and bottom observations of cell infiltration. In Fig. 6D_1_-D_6_**_1_-D_,_** we present SEM micrographs of scaffolds after 7 days of cell incubation with a focus on the top (Fig. 6D_1_-D_3_), and the bottom (Fig. 6D_4_-D_6_) of the scaffold. In all scaffolds, cells were visibly growing on the top surfaces of the fibers. PLLA scaffolds show a dense layer of cells on the top, while no cell is on the bottom of the scaffold. Minimal or no cellular infiltration beyond the surface layer of PLLA fibers suggests a barrier effect likely caused by the structural density of the fibers limiting cell migration through the scaffold. In contrast, analyzing together results from CLSM cell morphology and SEM, we observe a less dense cell layer on the top of PLLA-rGO and PLLA-Ti_3_C_2_T_*x*_ scaffolds. Still, there is a higher degree of cell infiltration demonstrated by the presence of cells at the bottom of these two samples, highlighted with red arrows (Fig. 6D_5_, D_6_). Definitely, the best infiltration was observed for PLLA-Ti_3_C_2_T_*x*_ with the highest number of cells on the bottom side. It can be assumed that both rGO and Ti_3_C_2_T_*x*_ incorporated in fibers affected the surface potential of fibers, which also led to changed scaffold geometry supporting better cellular penetration. rGO incorporated in fibers affected scaffold architecture due to large filler agglomerates. However, it must be noted that it is PLLA-Ti_3_C_2_T_*x*_ that shows the highest surface potential measured with KPFM and the highest level of cell infiltration. The highly charged PLLA-Ti_3_C_2_T_*x*_ fibers were repelling each other, increasing scaffold porosity and supporting better cell infiltration [[110], [111], [112]].

Previous studies have demonstrated that incorporating rGO into electrospun scaffolds can enhance cell proliferation, osteogenic differentiation, and even impart antimicrobial properties due to improved surface roughness and conductivity [[113], [114], [115]]. Similarly, MXene-containing scaffolds have shown potential for promoting protein adsorption, biomineralization, and supporting osteoblast activity, as reported by Awasthi et al. [39]. However, these studies did not directly evaluate or compare the impact of rGO and MXene fillers on local surface charge or correlate these effects with focal adhesion dynamics. Our study uniquely addresses this gap by analysis of surface charges and zeta potential of scaffolds and cells responses. This is a unique comparative approach to investigation of rGO and MXenes fillers in fibrous PLLA scaffolds. A summary of the key findings of our studies and their relevance for tissue engineering applications is provided in Table 5.

**Table 5:** Summary of the key findings from this study and their implications for tissue engineering applications. The table highlights the effects of rGO and Ti_3_C_2_T_*x*_ incorporation on scaffold properties and cell behavior.

Parameter Studied	Key Finding	Implications for Tissue Engineering
filler type (rGO vs Ti_3_C_2_T_*x*_)	Ti_3_C_2_T_*x*_ produced higher charged surfaces compared to rGO	filler selection affects bioelectric interactions with cells
fiber morphology	filler aggregates affected morphology and the mechanical performance of scaffolds	morphological and mechanical properties affect cell behavior and integration with scaffold
surface potential (KPFM)	PLLA-TI_3_C_2_T_*x*_ scaffolds showed highest surface potential	high surface potential promotes stronger/enhanced cell-material interactions
early cell adhesion (up to 5 h *in vitro* studies)	addition of rGO promoted early adhesion; PLLA-TI_3_C_2_T_*x*_ showed comparable adhesion to pure PLLA	early cell adhesion can affect subsequent cell responses
focal adhesion cluster size analysis	addition of TI_3_C_2_T_*x*_ increased cluster size and density of focal adhesion sites of cells	enhanced adhesion clusters can promote better integration of cells with scaffold
cell infiltration depth	cell infiltration improved from ∼70 μm (PLLA) to ∼100 (PLLA-rGO) and ∼125 μm (PLLA-TI_3_C_2_T_*x*_)	deep cells infiltration in the scaffolds improves and speeds up tissue regeneration
scaffold porosity and spacing between fibers	repelling effect and agglomeration of fillers led to more open structure of PLLA-TI_3_C_2_T_*x*_ scaffolds	scaffold 3D architecture can be tuned via surface charge effect on polymer fibers

## 4 Conclusions

This study demonstrates the consequences of the scaffold properties related to the incorporation of conductive fillers, such as rGO and Ti_3_C_2_T_*x*_, into electrospun PLLA fibers. We identified two major findings. First, the correlation between cell adhesion to fibers and cell infiltration into scaffolds is not straightforward. Second, the conductivity of the polymer solution during electrospinning, along with the surface charge retained by the produced fibers, has a significant impact on cellular responses in tissue engineering.

In more detail, the morphological analysis of fibers using CLSM revealed distinct agglomeration patterns where rGO forms larger and more variable clusters, while Ti_3_C_2_T_*x*_ produces smaller, more uniform agglomerates. These differences have significant implications for the mechanical performance of the fibers. Incorporating rGO and Ti_3_C_2_T_*x*_ into PLLA significantly altered the tensile strength, which decreased by 55 % with rGO and 81 % with Ti_3_C_2_T_*x*_ compared to neat PLLA. Toughness dropped substantially by 57 % with rGO and 88 % with Ti_3_C_2_T_*x*_, reflecting a significant reduction in the material's ability to absorb deformation energy. Additionally, strain at maximum stress remained stable for PLLA-rGO but was reduced by 90 % for PLLA-Ti_3_C_2_T_*x*_, indicating increased brittleness.

KPFM analysis revealed substantial shifts in surface potential, highlighting the impact of the fillers on fiber surface charge density. PLLA-rGO fibers exhibited a modest surface potential difference compared to PLLA (∼50 mV), while PLLA-Ti_3_C_2_T_*x*_ fibers showed a significant shift exceeding 400 mV. These surface potential changes correlate with the filler's influence on cellular interactions despite their sub-surface embedding, confirmed by the XPS analysis.

Cell studies showed that cell adhesion after 5 h of incubation is significantly better for PLLA-rGO than PLLA fibers. Later, cell focal adhesion points were verified, highlighting that the incorporation of rGO and Ti_3_C_2_T_*x*_ altered adhesion site architecture. Ti_3_C_2_T_*x*_ promoted the formation of larger, elongated, and denser adhesion clusters, while rGO enhanced the density of localized adhesion points. These results emphasize the role of filler type and distribution in modulating cell adhesion characteristics. Moreover, cell infiltration was affected by differences between scaffolds. Pure PLLA fibers support surface-level cell proliferation but exhibit limited cellular penetration, while the incorporation of rGO changes scaffold architecture and provides more cell anchoring spots, enabling deeper cell migration (∼100 μm). Ti_3_C_2_T_*x*_ also changes the geometry of the scaffold similarly to rGO but improves infiltration even better, allowing cells to penetrate uniformly to depths of ∼120 μm.

Importantly, incorporating rGO and Ti_3_C_2_T_*x*_ into electrospun PLLA fibers distinctly alters fiber morphology, mechanical properties, surface potential, and cellular behavior. While rGO creates the most diversified scaffold architecture and improves adhesion density, Ti_3_C_2_T_*x*_ stands out for its ability to enhance cellular infiltration and support extensive adhesion cluster formation. Despite the promising results presented in this study, certain limitations should be acknowledged. While versatile and scalable, the electrospinning process still faces challenges in achieving precise control over filler dispersion and maintaining batch-to-batch consistency. Additionally, isolating the specific contribution of surface charge from other properties, such as topography and stiffness, remains complex. Using non-degradable conductive fillers like rGO and MXenes also raises questions about long-term biocompatibility and clearance, which require further investigation. Nevertheless, our study contributes to the fundamental understanding of how local surface properties of biomaterials affect cell adhesion and infiltration. These insights are relevant for scaffold design in tissue engineering and provide a broader framework for interpreting cell–material interactions across various biomedical applications.

## CRediT authorship contribution statement

**Martyna Polak:** Writing – review & editing, Writing – original draft, Visualization, Validation, Methodology, Investigation, Formal analysis, Data curation, Conceptualization. **Krzysztof Berniak:** Writing – review & editing, Writing – original draft, Visualization, Methodology, Investigation. **Piotr K. Szewczyk:** Writing – review & editing, Writing – original draft, Visualization, Methodology, Investigation. **Joanna Knapczyk-Korczak:** Writing – review & editing, Visualization, Methodology, Investigation. **Mateusz M. Marzec:** Writing – review & editing, Visualization, Methodology, Investigation. **Muhammad Abiyyu Kenichi Purbayanto:** Writing – review & editing, Methodology, Investigation. **Agnieszka M. Jastrzębska:** Writing – review & editing, Supervision, Resources, Methodology. **Urszula Stachewicz:** Writing – review & editing, Writing – original draft, Visualization, Validation, Supervision, Resources, Project administration, Methodology, Funding acquisition, Formal analysis, Conceptualization.

## Notes

The authors declare that they have no known competing financial interests or personal relationships that could have appeared to influence the work reported in this paper. The raw/processed data required to reproduce this finding are available on request.

## Declaration of competing interest

The authors declare that they have no known competing financial interests or personal relationships that could have appeared to influence the work reported in this paper.

## Data Availability

Data will be made available on request.
