# Supplementary material for: Modulating cell adhesion and infiltration in advanced scaffold designs based on PLLA fibers with rGO and MXene (Ti3C2Tx)
Source: Mater Today Bio. 2025 Apr 21;32:101785. doi: 10.1016/j.mtbio.2025.101785 (PMC12059716; doi:10.1016/j.mtbio.2025.101785)
Supplement: Multimedia component 1 [file mmc1.docx]

**Supporting Information to:**

**Modulating Cell Adhesion and Infiltration in Advanced Scaffold Designs based on PLLA Fibers with rGO and MXene (Ti₃C₂T*_x_*)**

Martyna Polak^1^, Krzysztof Berniak^1^, Piotr K. Szewczyk^1^, Joanna Knapczyk-Korczak^1^, Mateusz M. Marzec^2^, Muhammad Abiyyu Kenichi Purbayanto^3^, Agnieszka M. Jastrzębska^3^, Urszula Stachewicz^1,*^

^1^Faculty of Metals Engineering and Industrial Computer Science, AGH University of Krakow, Al. A. Mickiewicza 30, 30-059 Krakow, Poland

^2^Academic Centre for Materials and Nanotechnology, AGH University of Krakow, Al. A. Mickiewicza 30, 30-059 Krakow, Poland

^3^Warsaw University of Technology, Faculty of Mechatronics, św. A. Boboli 8, 02-525 Warsaw, Poland

*E-mail: [ustachew@agh.edu.pl](mailto:ustachew@agh.edu.pl)

**
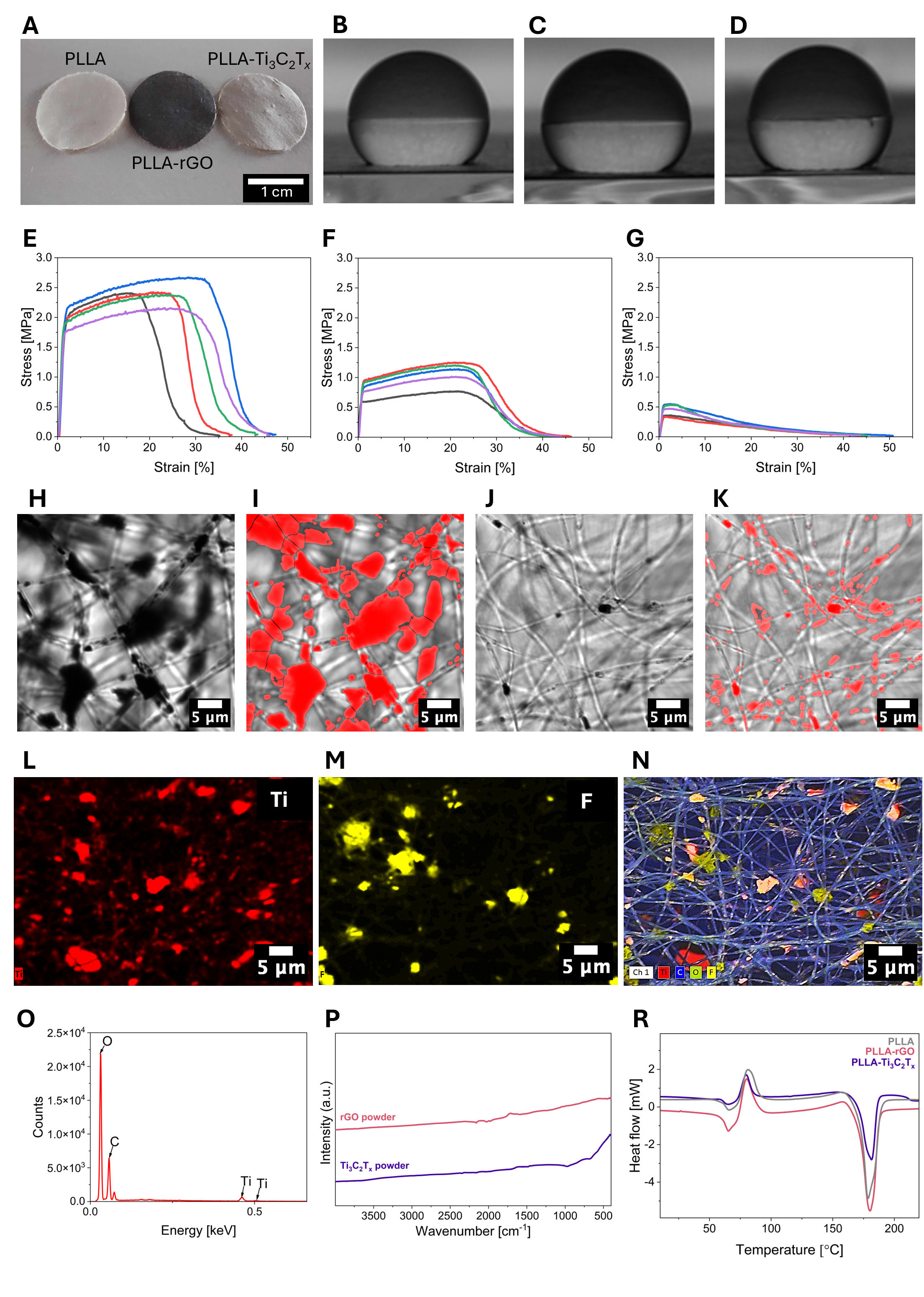
Figure S1.** A) Macroscopic images of scaffolds deposited on baking paper. Exemplary images of 3 µL water droplets deposited on B) PLLA, C) PLLA-rGO, and D) PLLA-Ti_3_C_2_T*_x_* scaffolds for static contact angle measurements. Stress-strain curves for F) PLLA, G) PLLA-rGO, and H) PLLA-Ti_3_C_2_T*_x_*. H) CLSM (ESID) representative image of PLLA-rGO for identification of rGO agglomerations, and I) accompanying images with a red mask indicating rGO agglomerations. Similarly, J) CLSM (ESID) representative images of PLLA-Ti_3_C_2_T*_x_*_,_ and K) images with a red mask indicating Ti_3_C_2_T*_x_* agglomerations. SEM EDS maps of L) titanium, M) fluorine, and N) merged channels for the PLLA-Ti_3_C_2_T*_x_* fibers. O) SEM EDS spectrum for PLLA-Ti_3_C_2_T*_x_* fibers. E) FTIR spectrum for rGO and Ti_3_C_2_T*_x_* powders. F) DSC curve for all scaffolds.

**
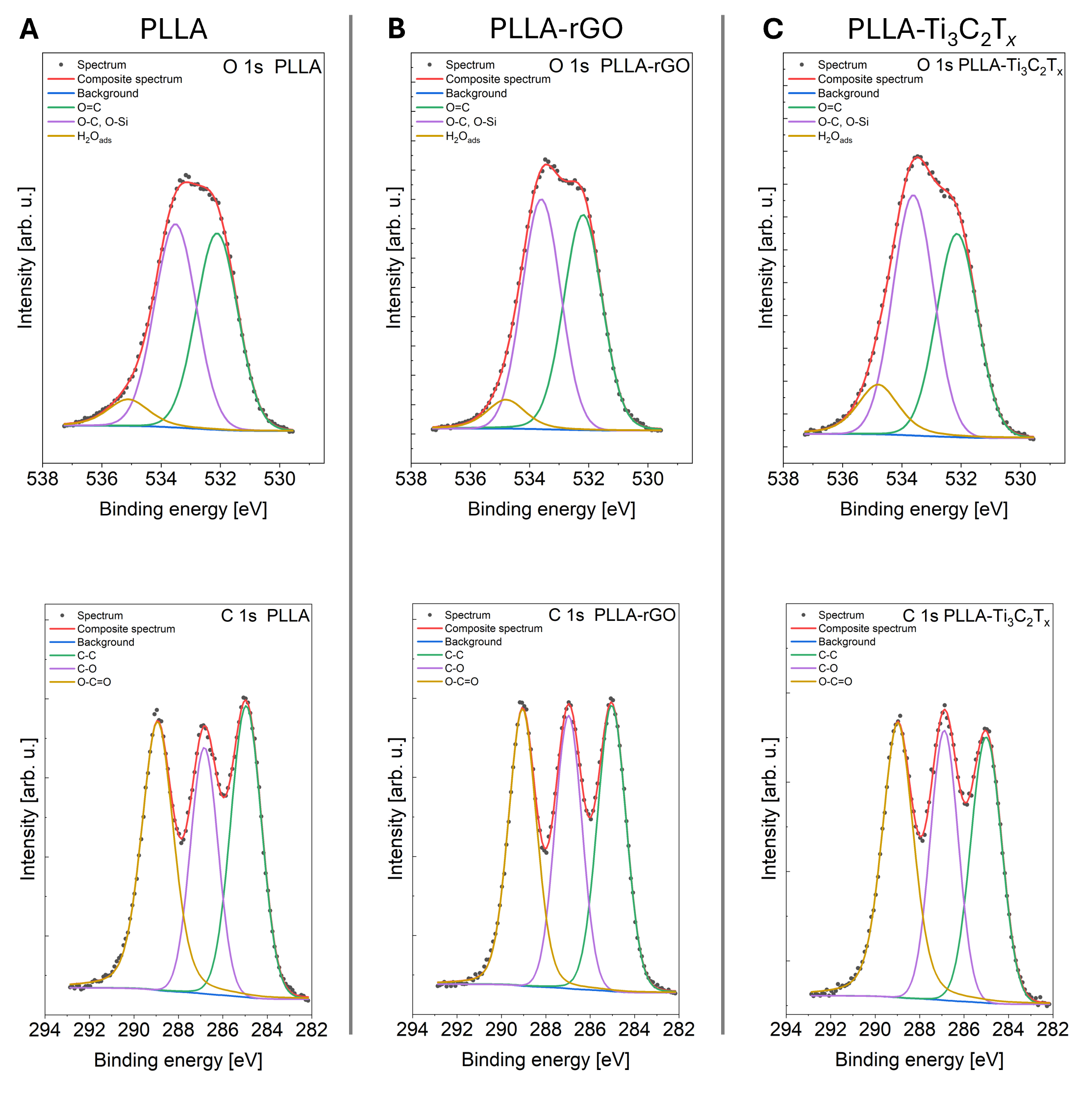
Figure S2.** High-resolution XPS spectra of A) PLLA, B) PLLA-rGO, and C) PLLA-Ti_3_C_2_T*_x_* scaffolds.

**
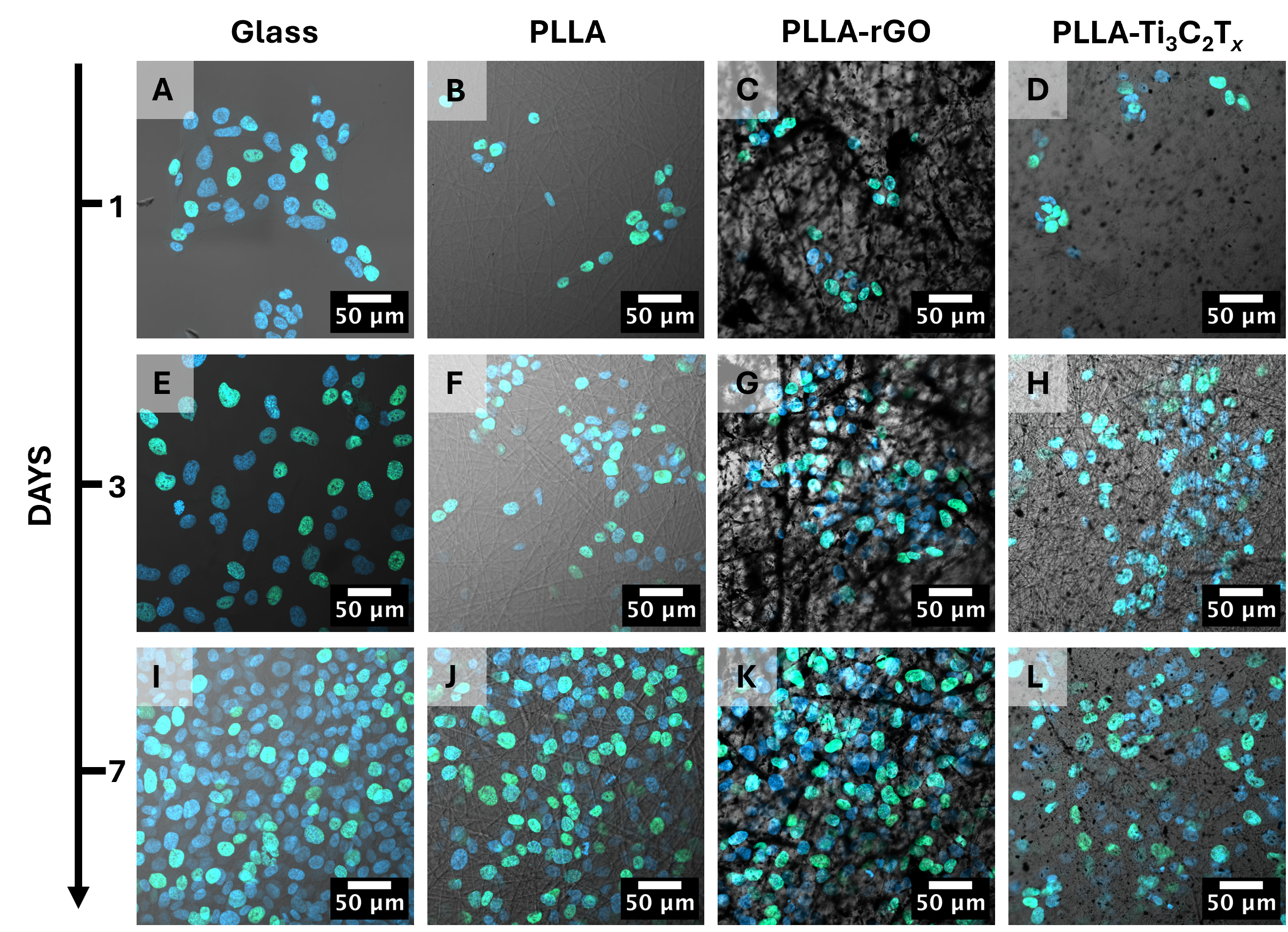
Figure S3.** CLSM micrographs representing osteoblasts on glass, PLLA, PLLA-rGO, and PLLA-Ti_3_C_2_T*_x_* scaffolds on day 1 (A–D), day 3 (E-H), and day 7 (I-L). Nuclei were stained with DAPI (blue); additionally, EdU incorporated during replication was stained with Alexa Fluor™ 488 (green).

**Movie S1.** Visualization of z-stack recorded with CLSM for PLLA, PLLA-rGO, and PLLA-Ti_3_C_2_T*_x_* scaffolds on the 7^th^ day of cell culture, presenting infiltration of cells starting from the top of the scaffold (defined as 0 µm imaging depth).
